# *Rickettsia typhi* and Haemophagocytic Syndrome

**DOI:** 10.4269/ajtmh.17-0606

**Published:** 2017-11-08

**Authors:** Chiara Iaria, Claudia Colomba, Paola Di Carlo, Francesco Scarlata, Manlio Tolomeo, Antonio Cascio

**Affiliations:** Infectious Diseases Unit -ARNAS Civico Di Cristina, Benfratelli Palermo, Italy E-mail: iaria.chiara@gmail.com; Department of Health Promotion Sciences and Mother and Child Care “G. D’Alessandro” University of Palermo Palermo, Italy E-mails: claudia.colomba@unipa.it, paola.dicarlo@unipa.it, francesco.scarlata@unipa.it, mtolomeo@hotmail.com, antonio.cascio03@unipa.it

Dear Sir,

We found the article by Pieracci et al.^1^ about Fatal Flea-Borne Typhus in Texas very interesting. However, the diagnosis of secondary hemophagocytic lymphohistiocytosis (HLH) should also have been considered.

HLH is a heterogeneous disorder that may be primary or secondary. The latter may be triggered by any severe infection, malignancy, or rheumatologic condition; it is diagnosed by five of eight of the following conditions: fever; splenomegaly; cytopenia (affecting ≥ 2 cell lineages); hypertriglyceridaemia and/or hypofibrinogenaemia; haemophagocytosis in the bone marrow, spleen, or lymph nodes; low or absent natural killer cell cytotoxicity; hyperferritinaemia; or elevated soluble CD25.^2^ HLH is considered a disorder due to a deficiency in cytolytic activity resulting in persistent activation of lymphocytes and histiocytes. This exaggerated inflammatory response is responsible for necrosis and organ failure and results in uncontrolled proliferation and phagocytic activity of histiocytes.^2^ We found only a few articles describing cases of HLH in patients with murine typhus,^3^ but many articles describe patients with severe or fatal forms of murine typhus in which a diagnosis of HLH should have been considered.

Animal studies on the pathogenesis of infection with *Rickettsia typhi*, the causative agent of murine typhus, have shown that *R. typhi* enters macrophages, the major cellular source of tumor necrosis factor α (TNFα), interleukin 6 (IL-6), and interleukin 12 (IL-12). IL-6 and TNFα are critical for rapid response to tissue injury and infections, and induce the production of acute phase reactants in the liver, whereas IL-12 is the main inducer of interferon γ in natural killer and T cells. This cytokine assists in bacterial killing by activating macrophage bactericidal functions. Death of *R. typhi*-infected CB17 severe combined immunodeficiency mice is most likely due to overwhelming systemic inflammation driven by macrophages and other cells.^4^

HLH is a life-threatening syndrome. Liver involvement may be present with variable levels of transaminitis progressing to acute liver failure and coagulopathy; respiratory insufficiency represents a negative prognostic sign. HLH can be triggered by rickettsial diseases.^5^ It should remembered that the identification of hemophagocytosis in bone marrow aspirates represents only one of the criteria needed for the diagnosis of HLH and that a bone marrow aspirate lacking hemophagocytosis does not rule out the diagnosis.^6^

HLH should be suspected in every patient with rickettsial diseases, especially with respiratory distress or multiorgan dysfunction. Appropriate therapy (dexamethasone, cyclosporin, and etoposide) could save the patient in those cases in which the pathogen-direct therapy has not been sufficient by itself to control the disease.
